# Uncovering Hidden Prognostic Patterns in Colorectal Cancer Histology Using Unsupervised Learning: A Computational Pathology Study

**DOI:** 10.3390/bioengineering13030334

**Published:** 2026-03-13

**Authors:** Wen-Tong Zhou, Yong Liu, Gang Yu, Kuan-Song Wang, Chao Xu, Jonathan Greenbaum, Chong Wu, Lin-Dong Jiang, Christopher J. Papasian, Hong-Mei Xiao, Hong-Wen Deng

**Affiliations:** 1 Center for System Biology, Data Sciences, and Reproductive Health, School of Basic Medical Science, Central South University, Changsha 410031, China; 2 Department of Biomedical Engineering, School of Basic Medical Science, Central South University, Changsha 410013, China; 3 Department of Pathology, Xiangya Hospital, School of Basic Medical Science, Central South University, Changsha 410078, China; 4 Department of Biostatistics and Epidemiology, University of Oklahoma Health Sciences Center, Oklahoma City, OK 73104, USA; 5 Tulane Center of Biomedical Informatics and Genomics, Deming Department of Medicine, School of Medicine, Tulane University, New Orleans, LA 70112, USA; 6 Department of Biostatistics, The University of Texas MD Anderson Cancer Center, Houston, TX 77030, USA; 7 Department of Biomedical Sciences, School of Medicine, University of Missouri-Kansas City, Kansas City, MO 64108, USA; 8 Institute of Reproductive & Stem Cell Engineering, School of Basic Medical Science, Central South University, Changsha 410000, China; 9 Key Laboratory of Biological, Nanotechnology of National Health Commission, Xiangya Hospital, Central South University, Changsha 410008, China

**Keywords:** colorectal cancer, histomorphological patterns, unsupervised learning, patient prognosis, pathological images

## Abstract

Colorectal cancer (CRC) remains a leading cause of cancer mortality globally, yet current histopathological diagnostics capture only limited features. This study aimed to discover subtle, prognostically significant histomorphological patterns in CRC tissues using unsupervised deep learning. We developed a framework integrating convolutional neural networks with deep clustering, trained on 23,341 image patches from 493 patients. We identified 30 distinct histomorphological clusters from CRC tissue images. Through univariate and multivariate survival analyses, three clusters (Cluster13, Cluster19, and Cluster24) were consistently associated with patient prognosis. These clusters were integrated with clinical factors (T stage, N stage, and differentiation degree) to construct a prognostic risk model. Patients stratified into high-risk and low-risk groups based on model predictions showed significant survival differences in both the training set (*N* = 493) and an independent validation set (*N* = 2590). Furthermore, logistic regression and multivariate Cox analyses demonstrated that incorporating the three histomorphological clusters alongside clinical factors yielded a modest but statistically significant improvement in predictive performance compared to clinical factors alone, indicating their complementary value for prognosis. This work demonstrates that computational pathology can uncover novel, visually elusive morphological features with independent prognostic value, offering potential to refine CRC patient stratification and inform clinical decision-making.

## 1. Introduction

The prevalence of Colorectal cancer (CRC), which is the third most common cancer (6.1%) and the second leading cause of cancer death (9.2%) worldwide, has increased rapidly in the past few decades [1]. In 2020, there were more than 1.9 million new cases of CRC and 935,000 deaths reported worldwide, and this number is expected to increase by at least 60% by 2030 [2,3]. Currently, histopathologic examination, where experienced pathologists visually inspect digital whole slide images (WSIs) of CRC tissue samples, remains the gold standard for CRC diagnosis. However, this diagnostic approach faces several challenges. These include the heterogeneity of cancer tissue histomorphology and the considerable time required for pathologists to analyze large slide images (>10,000 × 10,000 pixels). Furthermore, a global shortage of qualified pathologists exists. This shortage, combined with a growing workload, burdens many pathologists and may increase the risk of CRC misdiagnoses in routine histopathological examinations [4].

Histomorphological characteristics of CRC tissue are also critical for patient prognosis [5,6,7,8], but even experienced pathologists have difficulty analyzing tens of thousands of features in one WSI to identify and capture cancer patterns. Thus, pathologists have struggled to develop criteria for subdividing CRC’s into distinct patterns based solely on histomorphology of WSI [9]. Fortunately, data-driven artificial intelligence (AI) methods hold great promise for extracting and learning common cancer image features that can extract distinct histological features [10,11,12,13]. AI can analyze similarities in WSIs and detect subtle differences that may escape pathologists’ visual detection. These subtle features could contain crucial information for diagnosis and the prediction of clinical outcomes [5]. The subtle differences detected by AI can potentially be used to develop appropriate classification criteria for diagnosis and prognosis. Consequently, there is an urgent need to develop AI solutions that can assist pathologists in analyzing pathological images efficiently.

Current AI approaches, notably deep learning (DL), have exhibited remarkable capabilities in image processing. DL has been successfully applied to WSI analysis for several different cancer types, including lung [14,15], breast [16], lymph node [17,18], and skin [19] cancers. In our previous study, we proposed an AI-based method for CRC diagnosis using supervised learning (SL) to classify histopathological images. Using the largest dataset of labeled CRC WSI samples at that time, our method outperformed other AI methods applied to CRC diagnosis in previous studies [20]. Because we typically only have a small amount of labeled data and a much larger amount of unlabeled data in most real world situations, we recently developed a semi-supervised learning (SSL) method to diagnose CRC with a small amount of labeled data [21]. We demonstrated that the predictive accuracy of appropriately implemented SSL methods was comparable to that of experienced pathologists and to that of SL with massive labeled data [22]. However, there was one critical limitation. Both SL and SSL methods were focused on classifying histopathological images into CRC (cases) or non-CRC (controls) based on the annotations and labels for images that were determined by experienced pathologists. Thus, these approaches are not useful for generating new clinical knowledge from histopathological images, such as identifying hidden histopathological patterns.

In contrast, unsupervised learning (USL) holds great potential in identifying unknown cancer patterns. USL employs machine learning techniques, such as deep learning algorithms, to analyze and cluster unlabeled datasets [23]. This approach facilitates the discovery of hidden patterns or clusters without human intervention or bias. USL has been applied to various tasks, such as identifying novel radiomic subgroups [21], discovering novel biomarkers [24], and predicting treatment outcome [25] and survival [26]. For instance, different spatial or temporal sampling of tumors can have sub-populations of cells with distinct genomes, leading to visually distinct histological patterns [27,28]. Based on this observation, a previous study [29] applied a deep clustering convolutional autoencoder to classify cancer patterns for cholangiocarcinoma. The generated clusters were interpreted as new intrahepatic cholangiocarcinoma (ICC) patterns, and evaluated by survival analysis, resulting in statistically significant patient stratification for prognosis. A more recent study [30] proposed a new self-supervised learning approach to identify novel histopathological features and demonstrated that the identified histopathological features are statistically relevant for patient prognostic outcomes. One approach to USL, self-supervised learning, leverages the inherent structure or context of the data to generate its own labels or supervision signals, without relying on human-annotated labels [31]. Several other studies have focused on molecular patterns of CRC [32,33,34] and used deep learning of histopathological images to directly extract prognostic features from these patterns [35,36,37]. The advantages of USL-based approaches are that: (1) they eliminate the need for extensive manual work because they do not require annotation of images beforehand, and; (2) they can identify subtle morphologic differences between different regions within microscopic images, leading to the discovery of novel features that evade visual inspection of even the most experienced pathologists. Importantly, the potential clinical significance of these distinguishing features must be verified by subsequent analysis to validate their biological and/or clinical significance.

The primary objective of our research was to develop a USL-based approach to identify hidden histomorphological patterns in CRC tissues associated with patient prognosis. The histomorphological patterns are anticipated to serve as novel prognostic indicators, potentially improving treatment protocols and enhancing patient survival rates. Our investigation highlights the pivotal influence of computational pathology in propelling the future of disease prognosis and personalized medicine.

## 2. Materials and Methods

### 2.1. Study Design and Setting

**Figure 1:**
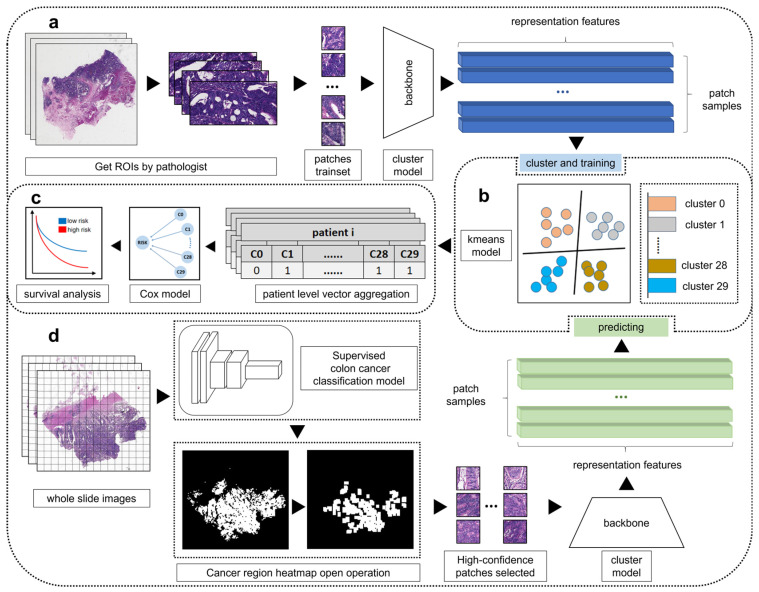
Study design and pipeline. Our approach consists of four major steps: (**a**) We trained a cluster representation learning network to extract effective features on the training dataset. (**b**) We used the k-means clustering algorithm to group the patches based on their visual similarity. (**c**) We aggregated the patch clustering labels to patient level vectors. Then we performed survival analyses with the patient level vectors to identify the clusters significantly associated with patient prognoses in training dataset. (**d**) Then we applied the trained clustering model to group these patches into distinct clusters. Survival analysis were also performed on validation cohort to validate the association between histomorphological patterns and patient prognoses.

In this study, we proposed a four-stage pipeline to obtain and validate histomorphological patterns of CRC as shown in Figure 1. Our study consists of four major steps:

a. We trained a cluster representation learning network to extract effective features on the training dataset.
b. We used the k-means clustering algorithm to group the patches based on their visual similarity.
c. We aggregated the patch clustering labels to patient level vectors. Then we performed survival analyses with the patient level vectors to identify the clusters significantly associated with patient prognoses in training dataset.
d. Then we applied the trained clustering model to group these patches into distinct clusters. Survival analyses were also performed on validation cohort to validate the association between histomorphological patterns and patient prognoses.

### 2.2. Patient Cohorts and Clinical Information

As shown in Table 1, we collected two cohorts of CRC patients from the Xiangya Hospital (Central South University in Changsha City, Hunan Province, China) for this study. The first cohort, consisting of 493 patients with diagnostic WSIs, was designated as the training set (Xiangya Hospital-Training set, or XYH-T set). For the first cohort, pathologists manually segmented the regions that only contained cancerous tissues from the diagnostic WSIs of 493 patients as the representative regions of interest (ROIs). The second cohort, XYH-PV set (Xiangya Hospital-Patient Validation set), consisting of 2590 patients with diagnostic WSIs but without manually segmented ROIs, was designated as the validation set (Xiangya Hospital-Patient Validation set, or XYH-PV set). The two cohorts were checked independently by two professional expert pathologists to ensure consistent diagnosis of the patients. There were no overlapped patients between the two cohorts, and no family related individuals included in either cohort. Thus, the two cohorts were independent of each other.

Clinical information for the two cohorts, including age, sex, tumor node metastasis (TNM) classification, histological type, survival status, cancer differentiation degree and survival time (i.e., time between diagnosis and death) were collected from the Xiangya hospital electronic information system. The TNM classification is a widely recognized standard for classifying the spread of malignant tumors [38]. The TNM system uses three categories to describe the extent of the cancer: T for the size and extent of the primary tumor, N for the number and location of nearby lymph nodes that have cancer, and M for the presence or absence of distant metastases. The T stage is usually determined by examining the tumor under a microscope or by using other imaging tests. The T stage is assigned a number from 0 to 4, depending on the size and location of the tumor; the higher the number, the larger or more invasive the tumor is. The N stage is a term used in cancer staging to describe how much the cancer has spread to nearby lymph nodes. The N stage is assigned a number from 0 to 3; the higher the number, the more advanced the cancer is. The degree of differentiation refers to the dissimilarity of tumor tissue to normal tissue morphology, which is usually divided into four levels (well differentiated, moderately differentiated, poorly differentiated and undifferentiated) [39]. Generally, well differentiated tumor cells have greater similarity to normal cells, and tend to grow slowly, metastasize less, and have a better prognosis. Poorly differentiated tumor cells have lower similarity to normal cells, and tend to grow rapidly, metastasize more, and have a worse prognosis. Due to too many missing values of M stage in the data, the M stage information was removed from the present study.

This study was a retrospective analysis of deidentified patient data from the Health Management Information System of Xiangya School of Medicine, Central South University. Due to the retrospective nature of the study and use of fully de-identified data, informed consent was waived by the Institutional Review Board (IRB) of Xiangya School of Medicine, Central South University.

### 2.3. Dataset Pre-Processing

The XYH-T set was manually annotated by professional pathologists for patch generation, while the XYH-PV set was only labeled at the WSI level. To maximize the confidence of filtrated cancer patches, the processing procedures were different for each cohort. For the XYH-T set, the ROIs were annotated by independent professional pathologists, and then tiled and segmented into non-overlapping patch images of 300 × 300 pixels. In total, the ROIs in XYH-T set were divided into 23,341 patches of cancerous regions. For the XYH-PV set, the ROIs were generated by applying a validated highly accurate supervised CRC recognition model [20]. Consistently, the ROIs were divided into non-overlapping patch images of 300 × 300 pixels. In total, the ROIs in XYH-PV set were divided into 7,744,176 patches.

### 2.4. Cluster Model

We applied the feature clustering method based on DeepCluster [40] and selected the Inception v3 [41] model as the backbone for training the feature extraction model. DeepCluster is a self-supervised learning approach for learning visual features from images without using any labels. It uses a convolutional neural network, such as Inception V3, to extract features from the images, and then used a k-means clustering algorithm to cluster them based on their similarity. The cluster assignments are then used as pseudo-labels to fine-tune the network parameters. By iteratively applying this process, DeepCluster can learn the parameters of a neural network and the cluster assignments of the resulting features. The model training processing is illustrated in Figure S1. Each training step consists of two stages: clustering stage and training stage. In the clustering stage, we extracted features from all patch images and reduced them to 32-dimension feature vectors by principal component analysis (PCA) [42]. We fit the model with a series of K’s from 5 to 50 and drew the normalized mutual information (NMI) [43] curve during training steps as in Figure S2. The NMI is a measure used in information theory and statistics to assess the similarity between two kinds of different clustering or classification of a dataset. Specifically, it normalizes the mutual information score to scale the results between 0 (indicating no mutual information) and 1 (representing perfect correlation). When the NMI curve is stable and maintains a high value, a smaller number of clusters is beneficial for prognostic analysis. We observed that the NMI curve was stable and maintained a high value (>0.8) when the number of clusters was at least 30. Thus, we assigned the number of clusters to 30 for our final model. In the training stage, we added a fully connected classification layer of size 30 to the backbone cluster model, which was trained and tuned based on the pseudo-labels via optimizing a cross entropy loss function [44]. To evaluate the cluster stability during model training, we calculated the NMI [43] between the current epoch and the previous epoch.

### 2.5. Patient Level Vector

A patch was defined as a 300 × 300-pixel image fragment extracted from tumor regions of WSIs, serving as the fundamental unit for subsequent histomorphological analysis. Each patient contributed multiple patches, capturing diverse morphological features across their tumor tissue. To enable patient-level survival analysis, the patch-level cluster assignments needed to be aggregated into a patient-level summary. We used a 30-dimension vector v to describe all the histomorphological characteristics for each patient, where each element indicates whether the corresponding histomorphological pattern occurred in the patient’s WSIs. V is used to represent the set of all vectors v at the patient level. The vector set V and vector v was described as follows:

(1) $$V=v_{1},\ldots ,v_{k}$$

(2) $$v_{k}=\left[e_{0},\ldots e_{i,\;i\in \left[0,29\right]}\right]$$

(3) $$e_{i}=\left\{\begin{array}{c} 1,\;if\;S_{i}\;in\;I_{k}\\ 0,\;else\; \end{array}\right.$$

where V is a set of patient level vectors, composed of k patients. Each patient is represented by a vector $v_{k}$ of 30 dimensions. For the kth patient, $S_{i}$ represents the image belonging to the cluster i, while $I_{k}$ represents all the pathological images belonging to the patient k. In each patient’s vector v, a value of 1 indicates that the corresponding histomorphological pattern occurred in the patient’s WSI, while a value of 0 indicates the opposite (not occurred). In this way, we extracted all image features, assigned cluster labels to each patch image, and aggregated patch image labels to patient level vectors.

### 2.6. Statistical Analysis

In this study, we first performed univariate Cox proportional hazards regression analysis for each histomorphological cluster in the XYH-T cohort to preliminarily identify clusters associated with overall survival. A total of 493 patients with CRC were included in the survival analysis, with 96 death events recorded during follow-up. Clusters with a *p*-value < 0.05 were considered statistically significant, and their hazard ratios (HRs) with 95% confidence intervals (CIs) were calculated. Considering potential clinical relevance and collinearity among these clusters, we then incorporated the significant clusters along with clinical covariates—including gender, clinical stage (T1, T2, T3, T4, N0, N1 and N2), and differentiation degree (poorly, moderately or well differentiated)—into a multivariate Cox regression model. Variables that retained statistical significance (*p* < 0.05) in the multivariate analysis were regarded as independent prognostic factors.

In parallel, we applied least absolute shrinkage and selection operator (LASSO) Cox regression, incorporating all 30 clusters and the clinical variables simultaneously, to perform data-driven feature selection. The optimal penalty parameter λ was determined by 10-fold cross-validation based on the partial likelihood deviance, and variables with non-zero coefficients were selected.

By integrating the results from both approaches, we selected the final set of clinical variables and clusters for constructing the prognostic risk model. For each selected histomorphological cluster, we performed univariate Kaplan–Meier survival analysis (without clinical factors) to compare survival distributions between patients with and without the cluster. These analyses were conducted in both the XYH-T (*N* = 493) and XYH-PV (*N* = 2590) datasets, and differences were assessed using the log-rank test. HRs and their 95% CIs were calculated for all predictors in the Cox regression model, and model performance was evaluated using the concordance index (C-index). Survival times were measured in years, and a common truncation time of 4.6 years (the maximum follow-up in the training set) was applied to both datasets to ensure a consistent observation window for the analyses. Next, we constructed a multivariate Cox model in the XYH-T set that included the clinical variables (T status, N status and differentiation degree) together with the selected histomorphological clusters.

Using the final prognostic model constructed from the XYH-T training cohort, we first calculated risk scores for all patients in both the XYH-T (*N* = 493) and XYH-PV (*N* = 2590) datasets. Patients were then stratified into high-risk and low-risk groups based on the median risk score derived from the XYH-T cohort. Kaplan–Meier survival analysis was performed to compare survival differences between the two risk groups in both datasets. To further evaluate the validity of this risk stratification, we assessed the net benefit and time-dependent predictive accuracy in each cohort.

To further investigate whether incorporating histomorphological patterns alongside clinical information improves survival prediction, we conducted additional analyses in both the XYH-T (*N* = 493) and XYH-PV (*N* = 2590) datasets. First, using 4.6-year survival status as the endpoint, we applied logistic regression to compare models with and without the histomorphological patterns. Predictive performance was assessed using the area under the receiver operating characteristic curve (AUC), and AUCs were compared using DeLong’s test. Second, using 4.6-year as the censoring threshold, we performed multivariable Cox regression to evaluate the added value of the histomorphological patterns. Finally, we calculated the continuous net reclassification improvement (NRI) and integrated discrimination improvement (IDI) to quantify the incremental predictive benefit of incorporating histomorphological patterns beyond clinical factors alone.

We performed all the statistical analyses using R software (version 4.1.1). Specifically, the survival analyses were performed using the ‘rms’ (version 6.7), ‘survival’ package (version 3.2), ‘survminer’ package (version 4.1.2) and ‘glmnet’ package (version 4.1.10). AUCs and the 95% confidence interval (CIs) were computed using the ‘pROC’ package (version 1.18). The continuous NRI was defined as the sum of NRI^+^ (events correctly reclassified upward) and NRI^−^ (non-events correctly reclassified downward). The IDI was computed as the difference in mean predicted risks between the full and clinical-only models for events minus that for non-events. The 95% CIs of NRI and IDI were obtained via bootstrap resampling with 1000 iterations, with the R package ‘nricens’ (version 1.6). We implemented the deep learning code in Python (version 3.8) with the PyTorch framework (version 1.8.1) and the torchvision library (version 0.9.1).

## 3. Results

### 3.1. Performance of the Clustering Model

Using the deep learning cluster model, all patches were grouped into 30 clusters. The model training process was described in the Methods, and the cluster model became stable after the 20th epoch. In subsequent training, the NMI value of the model fluctuated around 0.8, indicating that minor adjustments were needed in some samples for each cluster, and thus stable clusters were achieved in the later training period Figure S3.

**Figure 2:**
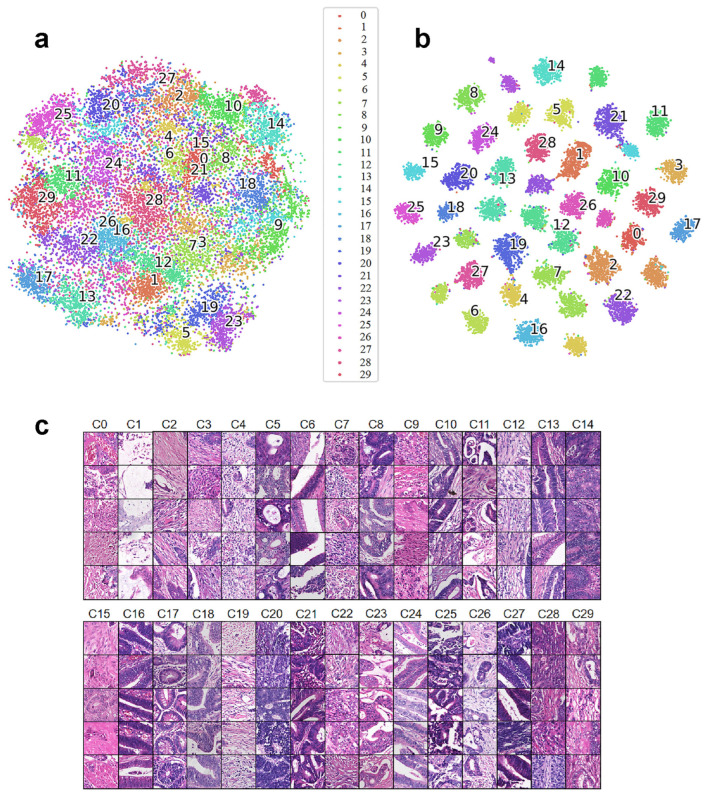
Visualization of the clustering effect with the t-distributed stochastic neighbor embedding (TSNE) and cluster sample. The numbers represent the codes corresponding to the clusters. (**a**) Clustering result at the 10th epoch. (**b**) Clustering result at the 199th epoch. (**c**) Visualization of the clustering effect with the actual image. For each cluster, we randomly selected five images. The images within each cluster showed high histomorphological similarity, indicating that the model effectively captured the histomorphological patterns in the histopathologic images of CRC.

To visualize the distribution of derived clusters, we performed t-distributed stochastic neighbor embedding (TSNE) to extract the two most representative dimensions. To visualize the clustering results, we also randomly selected patch images from each histomorphological pattern to visually examine their histomorphological similarity. As shown in Figure 2a, at the initial stage of training (after the 10th epoch), no clear histomorphological patterns were formed among the patches, and all images were mixed together. In contrast, as shown in Figure 2b, at the end of training (after the 199th epoch), compact and distinct clusters were developed, indicating the effectiveness of model training and clustering.

To demonstrate the performance of the clustering model, we randomly sampled 5 images from each cluster. As shown in Figure 2c, the images within each cluster exhibited high histomorphological similarity, indicating that the model effectively captured the histomorphological patterns in the histopathologic images of CRC. The classified patches were shown to an experienced pathologist for validation. The pathologist confirmed that patches within the same clusters shared histomorphological similarity. However, these patterns did not correspond to pre-existing histological features, such as serrated morphology, mucinous differentiation, or invasive margins [45,46,47]. This indicates that our clustering model learned previously unknown histomorphological patterns.

### 3.2. Clusters Associated with Patient Prognosis

The DeepCluster algorithm successfully partitioned the histopathological images into 30 distinct clusters based on inherent morphological similarities. To investigate whether the clustering of CRC is clinically relevant, we aggregated cluster labels of each patch to form a set of CRC vector features for each patient, where the vector indicates the occurrence or non-occurrence of each cluster in the patient. Then we performed survival analyses at the patient level (*N* = 471 after excluding individuals with missing data; with 96 death events) to identify clusters significantly correlated with the patient survival time (i.e., time between CRC diagnosis and death).

We first performed univariate Cox regression analysis for each of the 30 clusters in the XYH-T cohort. Six clusters showed a nominal significant association with overall survival at *p* < 0.05: Cluster8 (HR = 0.57, 95% CI 0.37–0.90, *p* = 0.014), Cluster13 (HR = 0.53, 95% CI 0.36–0.80, *p* = 0.002), Cluster16 (HR = 0.64, 95% CI 0.42–0.97, *p* = 0.034), Cluster19 (HR = 1.99, 95% CI 1.30–3.07, *p* = 0.002), Cluster24 (HR = 0.59, 95% CI 0.39–0.89, *p* = 0.012), and Cluster26 (HR = 1.55, 95% CI 1.03–2.33, *p* = 0.035) (Table 2). To adjust for potential confounding, these six clusters were subsequently entered into a multivariate Cox model together with clinical covariates (gender, T stage, N stage, and differentiation degree). After adjustment, Cluster13 (HR = 0.64, 95% CI 0.42–0.99, *p* = 0.044), Cluster19 (HR = 2.31, 95% CI 1.47–3.65, *p* = 3.13 × 10^−4^), and Cluster24 (HR = 0.54, 95% CI 0.35–0.86, *p* = 0.009) retained independent prognostic significance (Table 2). To verify that the clusters included in the Cox model satisfy the proportional hazard assumption, which expects the HR to remain constant over time, we performed the Schoenfeld residual test [48]. As shown in Figure S4, the HR of the significant clusters (Cluster13, Cluster19 and Cluster24) did not change over time (*p* > 0.05), supporting the validity of our Cox regression analysis. In addition, we applied LASSO Cox regression incorporating all clusters and clinical variables to perform data-driven feature selection. The LASSO model identified non-zero coefficients for the following variables: Cluster1, Cluster2, Cluster13, Cluster15, Cluster19, Cluster20, Cluster24, T2 stage, N2 stage, and poor differentiation degree (Table 2).

We constructed the final multivariate Cox model by integrating variables selected from both multivariate Cox regression (adjusting for clinical factors) and LASSO Cox regression. The model included T stage, N stage, differentiation degree, and three histomorphological clusters: Cluster13, Cluster19, and Cluster24. As shown in Table 3, after adjustment, Cluster19 was confirmed as a significant risk factor (HR = 2.38, 95% CI 1.52–3.72, 1.54 × 10^−4^), while Cluster13 (HR = 0.64, 95% CI 0.42–0.97, *p* = 0.037) and Cluster24 (HR = 0.50, 95% CI 0.32–0.76, *p* = 1.34 × 10^−3^) were associated with improved survival. The model demonstrated good discriminatory ability with a C-index of 0.72. The likelihood ratio test confirmed the overall significance of the model (*p* = 9 × 10^−11^). These results suggest that the identified histomorphological clusters provide prognostic information complementary to conventional clinical factors and may serve as complementary markers for risk stratification in CRC.

### 3.3. Prognosis Evaluation of Patients with Significant Clusters

**Figure 3:**
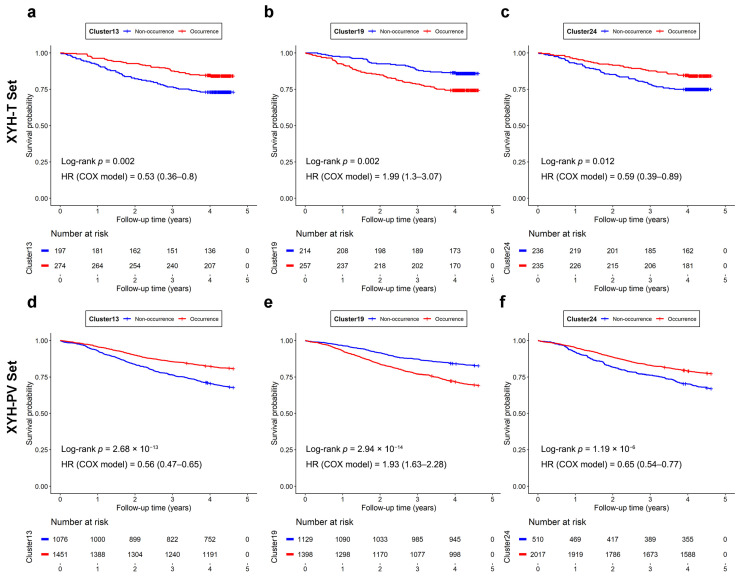
The univariate Kaplan–Maier survival analysis for each histomorphological cluster in the XYH-T (**a**–**c**) and XYH-PV (**d**–**f**) cohorts. (**a**,**d**) Cluster13, (**b**,**e**) Cluster19, (**c**,**f**) Cluster24. Patients were stratified by the occurrence or non-occurrence of the cluster. The log-rank test was used to compare survival curves between groups; hazard ratios (HRs) were estimated using univariate Cox regression.

To investigate the effect of the three significant clusters on prognostic evaluation, patients were divided into occurrence and non-occurrence groups based on the presence of each cluster. For each significant cluster, this grouping was applied to both the XYH-T and XYH-PV sets (*N* = 2527 after excluding individuals with missing data), and Kaplan–Meier survival curves were plotted for the two groups. The results were similar between the two cohorts, confirming the reproducibility of the prognostic stratification. For all three significant clusters, significant survival differences were observed between occurrence and non-occurrence groups in both the XYH-T and XYH-PV sets (Figure 3). For Cluster13 and Cluster24, occurrence was associated with a significantly better prognosis (higher survival probability) compared with non-occurrence. Conversely, occurrence of Cluster19 was associated with a significantly worse prognosis (lower survival probability).

**Figure 4:**
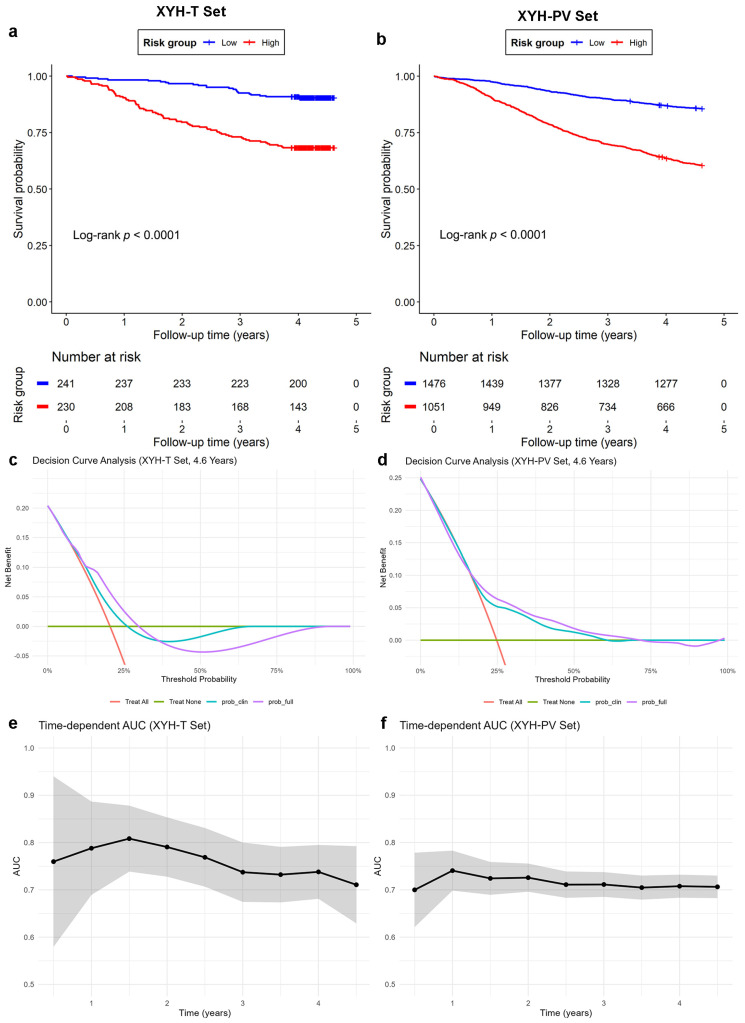
Prognostic value of the combined model incorporating the three histomorphological clusters and clinical factors. (**a**,**b**) Kaplan–Meier survival curves for high-risk versus low-risk groups in the XYH-T (**a**) and XYH-PV (**b**) sets. Risk groups were defined by the median risk score derived from the multivariate Cox model. (**c**,**d**) Decision curve analysis comparing the full model (clinical factors + clusters) with the clinical-factors-only model in the XYH-T (**c**) and XYH-PV (**d**) sets. (**e**,**f**) Time-dependent area under the curve (AUC) for the full model across 0.5 to 4.5 years in the XYH-T (**e**) and XYH-PV (**f**) sets. Shaded areas represent 95% confidence intervals.

To evaluate the combined prognostic value of the three histomorphological clusters, we constructed a multivariate Cox model in the XYH-T set that included the clinical variables (T status, N status and differentiation degree) together with Cluster13, Cluster19, and Cluster24. Based on this model, a risk score was calculated for each patient in both the XYH-T and XYH-PV set. Then the patients were stratified into high-risk and low-risk groups using the median risk score in the XYH-T set as the cutoff. The Kaplan–Meier curves showed that the low-risk group had significantly better survival than the high-risk group in both the XYH-T (Figure 4a) and XYH-PV (Figure 4b) sets, indicating the robustness of the combined predictors.

The clinical utility of the model was further assessed by decision curve analysis. In the XYH-T cohort, the full model (including both clinical factors and histomorphological clusters) provided a positive net benefit across clinically relevant threshold probabilities of approximately 13–30%, outperforming the clinical-factors-only model (Figure 4c). In the XYH-PV cohort, the full model maintained a positive net benefit over a broader threshold range (approximately 18–65%) and remained moderately superior to the clinical-factors-only model (Figure 4d). These findings indicate that the inclusion of the three histomorphological clusters enhances clinical utility, particularly in the moderate-risk range. Time-dependent predictive accuracy analysis demonstrated stable discriminative ability over time. In the XYH-T set, AUC values ranged from 0.71 to 0.81 between 0.5 and 4.5 years, with a peak of 0.81 at 1.5 years (Figure 4e). In the XYH-PV set, AUCs remained consistently between 0.70 and 0.74 across all time points (Figure 4f). These results confirm that the combination of clinical factors and three histomorphological clusters yields robust and time-stable prognostic discrimination.

**Figure 5:**
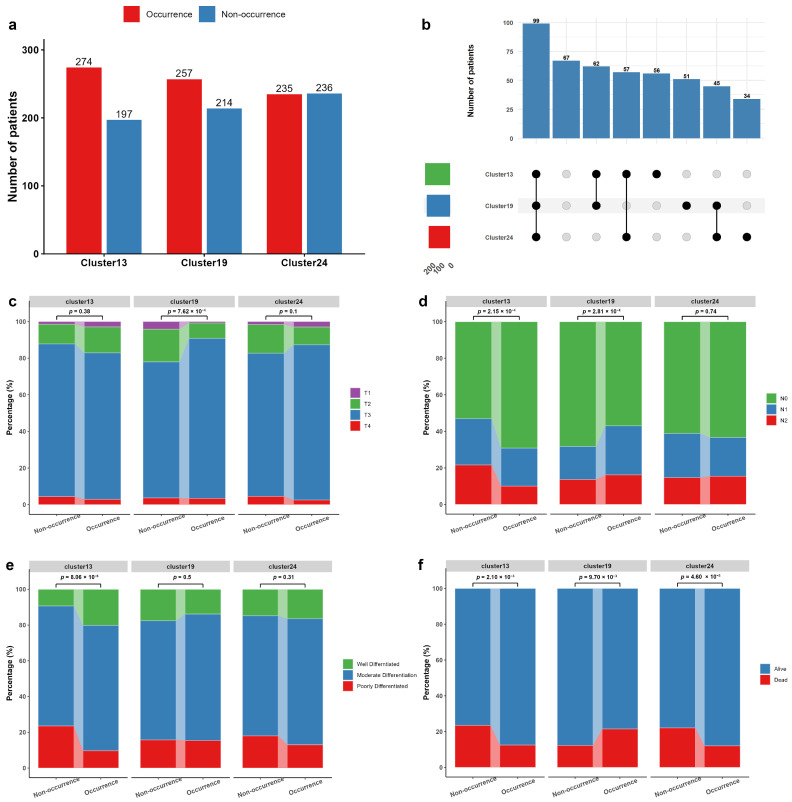
Distribution of the three prognostically significant histomorphological clusters and their associations with clinical factors and 3-year mortality in the XYH-T cohort. (**a**) Occurrence frequencies of Cluster13, Cluster19 and Cluster24. (**b**) UpSet plot illustrating the overlap among the three clusters. (**c**) Association of each cluster with T stage. (**d**) Association of each cluster with N stage. (**e**) Association of each cluster with differentiation grade. (**f**) Association of each cluster with 3-year mortality. *p* values were calculated using the Chi-square test.

We quantified the number of patients with and without each cluster; the complete counts for all clusters are provided in Table S1. Figure 5a shows the occurrence frequencies of the three prognostically significant clusters (Cluster13, Cluster19 and Cluster24) in the XYH-T set. Notably, these clusters were not mutually exclusive, and a considerable proportion of patients presented with two or more clusters simultaneously (Figure 5b). To examine whether the occurrence of the three clusters (Cluster13, Cluster19, and Cluster24) associated with the clinical indicators of T stage, N stage, and cancer differentiation degree, we performed chi-square tests to compare the distribution of these indicators among the occurrence and non-occurrence groups for each cluster in the XYH-T set. As shown in Figure 5c–e, patients with the Cluster13 tended to be more likely to be diagnosed as N0 stage (*p* value = 2.15 × 10^−4^) and their cancer differentiation degree was more likely to be well differentiated (*p* value = 8.06 × 10^−6^). Patients with the Cluster19 tended to be less likely to be diagnosed as T1/T2 stage (*p* value = 7.62 × 10^−4^) and N0 stage (*p* value = 2.81 × 10^−4^). The Cluster24 did not show any significant association with these indicators.

We also analyzed the survival status of all patients within 3 years and found that all of the three clusters were significantly associated with the death within 3 years (Figure 5d). A significantly lower proportion of patients with the Cluster13 (*p* value = 2.10 × 10^−3^) and Cluster24 (*p* value = 4.60 × 10^−3^) died within 3 years of diagnosis than those without, while a significantly higher proportion of patients with the Cluster19 (*p* value = 9.70 × 10^−3^) died within 3 years. These results were consistent with the survival analyses, confirming that the presence of Cluster13 or Cluster24 is associated with a favorable prognosis, while Cluster19 is associated with an unfavorable prognosis.

To further investigate whether the identified histomorphological patterns are helpful for prognostic predictions of patients’ survival time, we performed logistic regression (using 4.6-year mortality as the outcome) and Cox regression (with survival times censored at 4.6 years) in both the XYH-T and XYH-PV cohorts. For each model, we considered three models: (1) only including the clinical factors (T stage, N stage and cancer differentiation degree); (2) only including the three histomorphological patterns (Cluster13, Cluster19 and Cluster24); (3) including both the clinical factors and histomorphological patterns.

**Figure 6:**
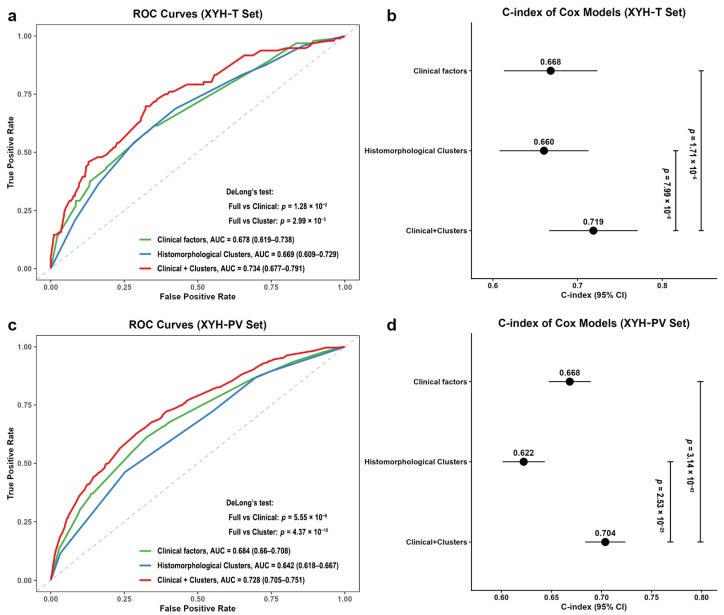
Performance of Logistic and Cox regression models. (**a**) Receiver operating characteristic (ROC) curves of the three Logistic models (clinical factors only, histomorphological clusters only, and their combination) in the XYH-T set. The dotted diagonal line represents the performance of a random classifier (AUC = 0.5), serving as a reference line. (**b**) C-index values of the three Cox models in the XYH-T set. The error bars show the 95% confidence intervals. (**c**) ROC curves of the three Logistic models in the XYH-PV set. (**d**) C-index values of the three Cox models in the XYH-PV set. *p* values for AUC comparisons were calculated using DeLong’s test; *p* values for C-index comparisons were derived from the likelihood ratio test.

In the XYH-T set, the logistic regression yielded AUCs of 0.678 (95% CI: 0.619–0.738) for clinical factors, 0.669 (95% CI: 0.609–0.729) for histomorphological patterns alone, and 0.734 (95% CI: 0.677–0.791) for the combined model. DeLong’s test showed that the combined model significantly outperformed both the clinical-only (*p* = 1.28 × 10^−2^) and cluster-only (*p* = 2.99 × 10^−3^) models (Figure 6a). Cox regression analyses showed consistent improvements. The C-index was 0.668 (95% CI: 0.613–0.723) for clinical factors, 0.660 (95% CI: 0.608–0.713) for histomorphological patterns alone, and 0.719 (95% CI: 0.666–0.771) for the combined model. Likelihood ratio tests confirmed that the combined model was superior to both the clinical-only (*p* = 7.99 × 10^−6^) and cluster-only (*p* = 1.71 × 10^−6^) models, even though the improvement in discrimination is moderate (Figure 6b). Similarly, in the XYH-PV set, the AUCs were 0.684 (95% CI: 0.660–0.708) for clinical factors, 0.642 (95% CI: 0.618–0.667) for clusters alone, and 0.728 (95% CI: 0.705–0.751) for the combined model, with *p* < 0.001 for both comparisons (DeLong test) (Figure 6c). The C-index values were 0.668 (95% CI: 0.647–0.689), 0.622 (95% CI: 0.601–0.643), and 0.704 (95% CI: 0.684–0.724) respectively, with *p* < 0.001 for both comparisons (Figure 6d).

We further evaluated the incremental value of the clusters using continuous NRI and IDI (Table 4). In the XYH-T set, the total NRI was 0.513 (95% CI: 0.295–0.728), with positive contributions from both events (NRI^+^ = 0.313, 95% CI: 0.118–0.489) and non-events (NRI^−^ = 0.200, 95% CI: 0.102–0.299). This indicates that the combined model correctly reclassified approximately 51.3% of patients. The IDI was 0.061 (95% CI: 0.035–0.087), reflecting a moderate improvement in the overall separation of risk predictions between died and alive patients. In the XYH-PV set, the total NRI was 0.388 (95% CI: 0.303–0.477), driven almost entirely by correct downward reclassification of non-events (NRI^−^ = 0.430, 95% CI: 0.388–0.471); the NRI^+^ was –0.042 (95% CI: –0.119–0.039). These findings suggest that the added value of the clusters in the validation set was primarily due to improved identification of low-risk patients. The IDI was 0.033 (95% CI: 0.023–0.043), reflecting a modest to moderate improvement.

These results demonstrate that integrating the three histomorphological patterns with clinical factors significantly improves discrimination and risk reclassification for CRC prognosis, and these improvements are validated in an independent cohort.

## 4. Discussion

CRC is a heterogeneous neoplastic disease [49,50] that exhibits diverse histomorphological patterns in tumor tissues. It is widely accepted that tumors have different regions with distinct morphological and molecular characteristics, which influence the tumor’s biological behavior and response to treatment [51]. By applying DL methods to analyze the histomorphological patterns of tissue slices, researchers have improved the prediction of survival outcomes for patients with endometrial, breast or other cancers [52,53]. These methods can extract hidden information related to survival from tissue slices, without relying on genetic testing or clinical indicators [14]. In the current study, we distinguished WSIs into different histomorphological patterns using AI, and explored the association of these patterns with survival time. This study aimed to identify hidden histomorphological patterns associated with CRC. We then sought to utilize these patterns to refine and improve prognostic stratification.

Three histomorphological patterns (Cluster13, Cluster 19 and Cluster24) identified by our approach were significantly associated with patient survival time after adjustment for clinical factor, such as T and N stages, indicating that they could serve as independent prognostic factors. Moreover, statistical analysis of the clinical information showed that two of these histomorphological patterns are associated with the existing clinical prognostic factors, such as T stage, N stage and degree of differentiation. In general, patients with T3/T4 stages, N1/N2 stages, and poorly differentiated cancer cells have a higher risk of cancer progression than those with T1/T2 stage, N0 stage, and well differentiated cancer cells [38,39]. We observed that a lower proportion of patients with occurrence of Cluster13 were characterized by high-risk factors, such as N1/N2 stages and poorly differentiated cancer cells, while a higher proportion of patients with occurrence of Cluster19 were characterized by high-risk factors. These results imply that these histomorphological patterns may be linked to the underlying molecular mechanisms of the tumor tissue that contribute to the patient’s clinical presentation, which warrants further functional investigation. Additionally, the models that integrated the clinical factors and histomorphological patterns showed higher performance, compared to clinical factors alone, in predicting of CRC patients’ survival time. These results indicate that the three identified histomorphological patterns can be used to provide additional independent information to supplement prognostications based on current clinical and pathologic criteria.

At present, the primary basis for the classification of CRC is molecular, and some molecular patterns of CRC have been shown to correlate with particular histological features, such as the serrated morphology, mucinous differentiation and invasive margin [50]. For instance, some CRCs have a serrated morphology, which means they have a jagged or star-shaped appearance when examined under a microscope. Serrated morphology is associated with certain molecular alterations, such as mutations in the Serine/Threonine-Protein Kinase B-Raf (BRAF) gene, CpG island methylator phenotype (CIMP), and microsatellite instability (MSI) [54,55,56]. Previous studies have shown that serrated morphology is associated with spread to the lymph nodes, reduced response to chemotherapy and poor prognosis in CRCs [57]. Mucinous differentiation is a feature of some CRCs that means some tumor cells can produce and secrete mucin, which is a slimy material that has glycoproteins and water [58]. According to the WHO criteria, a CRC is classified as mucinous adenocarcinoma (MAC) if more than 50% of the tumor is composed of extracellular mucin [59]. MAC is a distinct subtype of CRC that usually has a poorer prognosis and response to chemotherapy than non-mucinous CRC [58,60]. The invasive margin describes the shape and structure of the edge of a tumor that has grown into the surrounding normal tissue, which can indicate how aggressive the tumor is and how likely it is to spread to other parts of the body. The invasive margin can be classified as infiltrative, pushing, or mixed. It has been reported the invasive tumor margin can provide important prognostic information in CRC [45,46,47]. We showed the classified patches to an experienced pathologist, who confirmed that the patches in each cluster had similar histomorphological features. However, these features did not match the existing histomorphological patterns, suggesting that our clustering model discovered unknow histomorphological patterns.

A key aspect of this study is that we did not use any label information to develop the cluster model, such as cancer tissue labels, survival information, or clinical stage information. The feature extraction model was fully trained in an unsupervised way (independent of human knowledge and bias), and the resulting clusters were based solely on the histomorphological features of pathological images. Even without the guidance of relevant clinical information (e.g., disease stage), we obtained clusters that were associated with patient prognosis of survival via the unsupervised clustering model. These clusters have never been recognized by professional pathologists before this study, suggesting that the AI model may have discovered features that differ from currently acknowledged clinical histomorphologic patterns. This underscores the transformative role of USL-based approaches in advancing disease prognostication by bypassing the labor-intensive tasks of manual tissue labeling and exhaustive human evaluation of numerous histopathological images.

The primary goal of our study was to identify novel histomorphological patterns associated with patient survival, rather than to develop a clinically deployable risk model. It is important to emphasize that the identified clusters represent local tumor morphological patterns associated with prognosis, rather than defining new, distinct patient subgroups. These patterns reflect recurring histomorphological patterns present within tumor regions, and individual patients may harbor multiple such patterns simultaneously. Although the improvement in discrimination was moderate, the consistent selection of key histomorphologic patterns across multiple analytical approaches supports their ability to provide prognostic information that complements conventional clinical factors. In clinical practice, even modest gains in discrimination can contribute to better risk stratification when combined with existing clinical factors. More broadly, this work demonstrates a paradigm for integrating DL-based histomorphological analysis with prognostic modeling, highlighting the feasibility of this approach in medical image research. We believe that as larger, multi-institutional datasets become available and more comprehensive models are developed, such methods will increasingly extract robust and clinically meaningful information from routine pathological images. Ultimately, these advances may lead to more substantial improvements in prognostic stratification and risk assessment, bringing us closer to the goal of precision oncology.

Different types of cancer can have different histomorphological patterns, such as the size, shape, color, and arrangement of the cells [51]. These patterns can help doctors diagnose and classify cancers into distinct subtypes, which can have different outcomes and treatments. Previous studies have applied SL-based methods to classify cancer tissue slides into predefined categories and assessed prognosis of patients [61,62]. However, these approaches rely on pre-defined histological characteristics, such as tumor, stroma, veins, or inflammatory tissues. They may not extend readily to other cancer types where such prior knowledge or annotations are unavailable. In contrast, our USL-based framework automatically identifies and groups similar histomorphological features directly from cancer tissue images, without requiring predefined labels. Compared to a similar study in CRC, this study offers several advances. We trained our model on a substantially larger dataset, comprising 493 patients and over 23,000 tumor region patches. This allowed more comprehensive capture of histomorphological diversity. We further validated the identified clusters in a large independent cohort of 2590 patients, confirming the stability and robustness of the discovered histomorphological patterns. Additionally, we employed multiple complementary feature selection strategies, including univariate screening, multivariable Cox regression adjusting for clinical confounders, and LASSO Cox regression. These approaches consistently identified clusters associated with patient survival. By integrating these histomorphological patterns with routine clinical variables, we showed that they provide complementary prognostic information. Their inclusion led to a modest but statistically significant improvement in survival prediction compared to clinical factors alone. A distinct advantage of this approach is that it can be used to discover hidden patterns and histomorphological patterns of cancer that may not be obvious or known to human experts, even without any prior information or labels. Thus, the approach used in the current study is also applicable to other types of cancers. We hope that by identifying more hidden histomorphological patterns like this, we can achieve better grading of cancers and improve the treatment and survival outcome of patients.

Our study has some limitations: First, as we adopted a USL-based clustering model in this study, its generalization ability and performance may not be comparable to the supervised models. It may be challenging to directly apply the approach to the datasets from other hospitals or organizations, as the effectiveness of the feature extraction model may be influenced by some factors such as the differences in staining methods. By adjusting the model with local data, we can effectively reduce the impact of data bias. In this way, we can extend our approach to other cohorts and reveal additional population-specific or common histomorphological patterns of CRC. Second, the clinical information of the patients in this study was derived from existing medical records in the hospital, which might be incomplete or inaccurate. Although we applied strict selection criteria to improve data quality, potential biases cannot be entirely excluded. Furthermore, due to the retrospective nature of our study, certain clinically relevant variables—such as M stage—could not be incorporated into our analysis. For example, we did not include the M stage information in our study because of substantial missing data, and the M stage is an important factor affecting the patient’s prognosis. In future studies, we will prospectively collect this clinical information and incorporate them into our study, thereby improving our prediction performance for the patient’s prognosis. Third, the observed associations between histomorphological patterns and clinical characteristics should be interpreted as correlative rather than causal. Further studies incorporating molecular profiling and functional experiments are needed to uncover the underlying biological mechanisms. Fourth, the selection of 30 clusters was primarily guided by clustering stability rather than biological validation, and we cannot exclude the possibility that other cluster numbers might yield different survival associations. Determining the biologically optimal number of clusters ultimately requires deeper characterization of the underlying histomorphological features, which represents an important direction for future investigation.

## 5. Conclusions

By applying a USL-based approach, this study successfully identified three hidden histomorphological patterns associated with patient prognosis in CRC. These patterns have the potential to impact clinical management, leading to improved treatment strategies and patient outcomes. Our model introduces a novel perspective for identifying histological features with prognostic significance, bypassing the labor-intensive tasks of manual tissue labeling and exhaustive human evaluation of multiple histopathological images. These findings underscore the transformative role of computational pathology in advancing disease prognostication.

## Figures and Tables

**Table 1:** Baseline characteristics of the two cohorts.

Variable	XYH-T Set	XYH-PV Set
Number of Patients	493	2590
Number of Patches	23,341	7,744,176
Number of WSIs	493	5325
Age		
Mean ± Standard Deviation	62.82 ± 12.41	64 ± 12.25
0~60	214 (43.4%)	1021 (39.4%)
>60	279 (56.6%)	1569 (60.6%)
Gender		
male	301 (61.1%)	1565 (60.4%)
female	192 (38.9%)	1025 (39.6%)
Survival State		
alive	391 (79.3%)	1818 (70.2%)
dead	102 (20.7%)	772 (29.8%)
Degree of Differentiation		
Poorly Differentiated	75 (15.6%)	403 (15.9%)
Moderately Differentiated	332 (68.9%)	1538 (60.5%)
Well Differentiated	75 (15.6%)	601 (23.6%)
N Stage		
N0	306 (62.2%)	1489 (57.9%)
N1	112 (22.8%)	673 (26.2%)
N2	74 (15.0%)	411 (16.0%)
T Stage		
T1	11 (2.3%)	35 (1.4%)
T2	61 (12.7%)	413 (16.1%)
T3	391 (81.5%)	2069 (80.8%)
T4	17 (3.5%)	44 (1.7%)

**Table 2:** Cox regression analyses of the 30 clusters in the XYH-T set.

Univariate			Multivariate			LASSO	
Variable	HR	*p* Value	Variable	HR	*p*	Variable	Coefficient
Cluster0	0.92 (0.61–1.40)	0.706	Male	1.24 (0.81–1.91)	0.32	Male	0
Cluster1	1.51 (0.96–2.37)	0.071	T2	0.81 (0.10–6.83)	0.846	T2	−0.041
Cluster2	1.58 (0.95–2.64)	0.079	T3	1.47 (0.20–10.77)	0.706	T3	0
Cluster3	1.16 (0.78–1.74)	0.456	T4	1.66 (0.18–15.58)	0.656	T4	0
Cluster4	1.08 (0.71–1.62)	0.726	N1	1.11 (0.65–1.88)	0.702	N1	0
Cluster5	0.92 (0.61–1.39)	0.702	N2	3.40 (2.08–5.54)	9.25 × 10^−7^ ***	N2	0.977
Cluster6	1.15 (0.77–1.71)	0.498	Moderately Differentiated	0.87 (0.44–1.69)	0.674	Moderately Differentiated	0
Cluster7	1.15 (0.76–1.72)	0.514	Poorly Differentiated	1.64 (0.76–3.52)	0.206	Poorly Differentiated	0.496
Cluster8	0.57 (0.37–0.90)	0.014 *	Cluster8	0.78 (0.47–1.28)	0.322	Cluster0	0
Cluster9	1.20 (0.80–1.79)	0.389	Cluster13	0.64 (0.42–0.99)	0.044 *	Cluster1	0.057
Cluster10	0.74 (0.50–1.11)	0.144	Cluster16	1.04 (0.65–1.66)	0.865	Cluster2	0.118
Cluster11	1.45 (0.93–2.24)	0.101	Cluster19	2.31 (1.47–3.65)	3.13 × 10^−4^ ***	Cluster3	0
Cluster12	1.25 (0.81–1.92)	0.307	Cluster24	0.54 (0.35–0.86)	0.009 **	Cluster4	0
Cluster13	0.53 (0.36–0.80)	0.002 **	Cluster26	1.23 (0.81–1.87)	0.332	Cluster5	0
Cluster14	0.94 (0.62–1.43)	0.785				Cluster6	0
Cluster15	1.32 (0.88–1.98)	0.174				Cluster7	0
Cluster16	0.64 (0.42–0.97)	0.034 *				Cluster8	0
Cluster17	0.70 (0.46–1.05)	0.087				Cluster9	0
Cluster18	0.95 (0.60–1.51)	0.836				Cluster10	0
Cluster19	1.99 (1.30–3.07)	0.002 **				Cluster11	0
Cluster20	0.63 (0.37–1.06)	0.08				Cluster12	0
Cluster21	0.74 (0.49–1.12)	0.153				Cluster13	−0.219
Cluster22	1.32 (0.86–2.03)	0.201				Cluster14	0
Cluster23	1.15 (0.77–1.71)	0.499				Cluster15	0.109
Cluster24	0.59 (0.39–0.89)	0.012 *				Cluster16	0
Cluster25	1.21 (0.81–1.80)	0.353				Cluster17	0
Cluster26	1.55 (1.03–2.33)	0.035 *				Cluster18	0
Cluster27	0.96 (0.62–1.48)	0.852				Cluster19	0.496
Cluster28	0.80 (0.53–1.21)	0.289				Cluster20	−0.008
Cluster29	1.37 (0.92–2.04)	0.126				Cluster21	0
						Cluster22	0
						Cluster23	0
						Cluster24	−0.332
						Cluster25	0
						Cluster26	0
						Cluster27	0
						Cluster28	0
						Cluster29	0

* *p* < 0.05, ** *p* < 0.01, *** *p* < 0.001. Only clusters that were significant in univariate analysis (*p* < 0.05) were included in the multivariate Cox model. For categorical clinical variables, T1 was used as the reference for T stage, N0 for N stage, and well differentiation for differentiation degree.

**Table 3:** Cox regression analysis with significant clusters and clinical stages.

Variable		HR (95% CI)	*p*
Clinical factors	T2 (vs. T1)	0.70 (0.08–5.86)	0.742
	T3 (vs. T1)	1.28 (0.18–9.31)	0.809
	T4 (vs. T1)	1.38 (0.15–12.70)	0.774
	N1 (vs. N0)	1.11 (0.66–1.86)	0.706
	N2 (vs. N0)	3.44 (2.12–5.58)	6.08 × 10^−7^ ***
	Differentiation: Moderate (vs. Well)	0.88 (0.46–1.70)	0.702
	Differentiation: Poor (vs. Well)	1.75 (0.84–3.63)	0.135
Histomorphological clusters	Cluster13	0.64 (0.42–0.97)	0.037 *
	Cluster19	2.38 (1.52–3.72)	1.54 × 10^−4^ ***
	Cluster24	0.50 (0.32–0.76)	1.34 × 10^−3^ **
	Likelihood ratio test *p*-value = 9 × 10^−11^		
	C-index = 0.719 (se = 0.027)		

* *p* < 0.05, ** *p* < 0.01, *** *p* < 0.001.

**Table 4:** NRI and IDI for full model versus clinical factor-only model.

Dataset	Metric	Estimate	95% CI Lower	95% CI Upper
XYH-T	NRI	0.513	0.295	0.728
	NRI^+^	0.313	0.118	0.489
	NRI^−^	0.200	0.102	0.299
	IDI	0.061	0.035	0.087
XYH-PV	NRI	0.388	0.303	0.477
	NRI^+^	−0.042	−0.119	0.039
	NRI^−^	0.430	0.388	0.471
	IDI	0.033	0.023	0.043

NRI represents the net reclassification improvement; NRI^+^ represents the net reclassification improvement for events (events correctly reclassified upward); NRI^−^ represents the net reclassification improvement for non-events (non-events correctly reclassified downward); IDI represents the integrated discrimination improvement; CI represents the confidence interval.

## Data Availability

The datasets used and/or analyzed during the current study are available from the corresponding author on reasonable request.

## Supplementary Materials

The following supporting information can be downloaded at: https://www.mdpi.com/article/10.3390/bioengineering13030334/s1, Figure S1: The model training processing; Figure S2: Visualization of the normalized mutual information (NMI) curves during the training process with different numbers of clusters from 5 to 50; Figure S3: The normalized mutual information (NMI) curve during training. The maximum NMI was reached in epoch 132; Figure S4: Schoenfeld residual test for the 3 significant clusters. The top is Schoenfeld residual test of Cluster13. The middle is Schoenfeld residual test of Cluster19. The bottom is Schoenfeld residual test of Cluster24. All of the P values for the three clusters in Schoenfeld residual test were less than 0.05, indicating that the clusters included in the Cox regression satisfy the proportional hazard assumption; Table S1: Distribution of histomorphological clusters in the XYH-T and XYH-PV sets.

## Abbreviations

The following abbreviations are used in this manuscript:

AI	Artificial intelligence
AUC	Area under the receiver operating characteristic curve
BRAF	Serine/Threonine-Protein Kinase B-Raf gene
CI	Confidence interval
CIMP	CpG island methylator phenotype
C-index	Concordance index
Cox	Cox proportional hazards
CRC	Colorectal cancer
DL	Deep learning
HR	Hazard ratio
ICC	Intrahepatic cholangiocarcinoma
IDI	Integrated discrimination improvement
KM	Kaplan–Meier
LASSO	Least absolute shrinkage and selection operator
MAC	Mucinous adenocarcinoma
MSI	Microsatellite instability
NMI	Normalized mutual information
NRI	Net reclassification improvement
PCA	Principal component analysis
ROIs	Representative regions of interest
SL	Supervised learning
SSL	Semi-supervised learning
TNM	Tumor node metastasis classification
TSNE	T-Distributed Stochastic Neighbor Embedding
USL	Unsupervised learning
WSI	Whole slide image
XYH-PV	Xiangya Hospital-Patient Validation
XYH-T	Xiangya Hospital-Training
