# Supplementary material for: Uncovering Hidden Prognostic Patterns in Colorectal Cancer Histology Using Unsupervised Learning: A Computational Pathology Study
Source: Bioengineering (Basel). 2026 Mar 13;13(3):334. doi: 10.3390/bioengineering13030334 (PMC13024107; doi:10.3390/bioengineering13030334)

Supplementary Figures

Figure S1. The model training processing. In each epoch, the Inception V3 convolutional neural network was first applied to extract features from the input images. Principal component analysis (PCA) was then used to reduce the dimensionality of these features to 32 dimensions. Next, the images were clustered using the k-means method. Finally, the normalized mutual information (NMI) value of the clusters was calculated, and the weights of the network parameters were updated based on changes in NMI. The input images were scaled to 299×299 pixels to meet the requirements of the Inception V3 model and were randomly flipped to enhance generalization before being processed by the model. The learning rate was set as 1×10^{-4}, and the batch size was 128. The clustering model was trained on the XYH-T dataset for 200 epochs, using Adam optimizer with a weight decay of 1×10^{-5} and a momentum of 0.99.

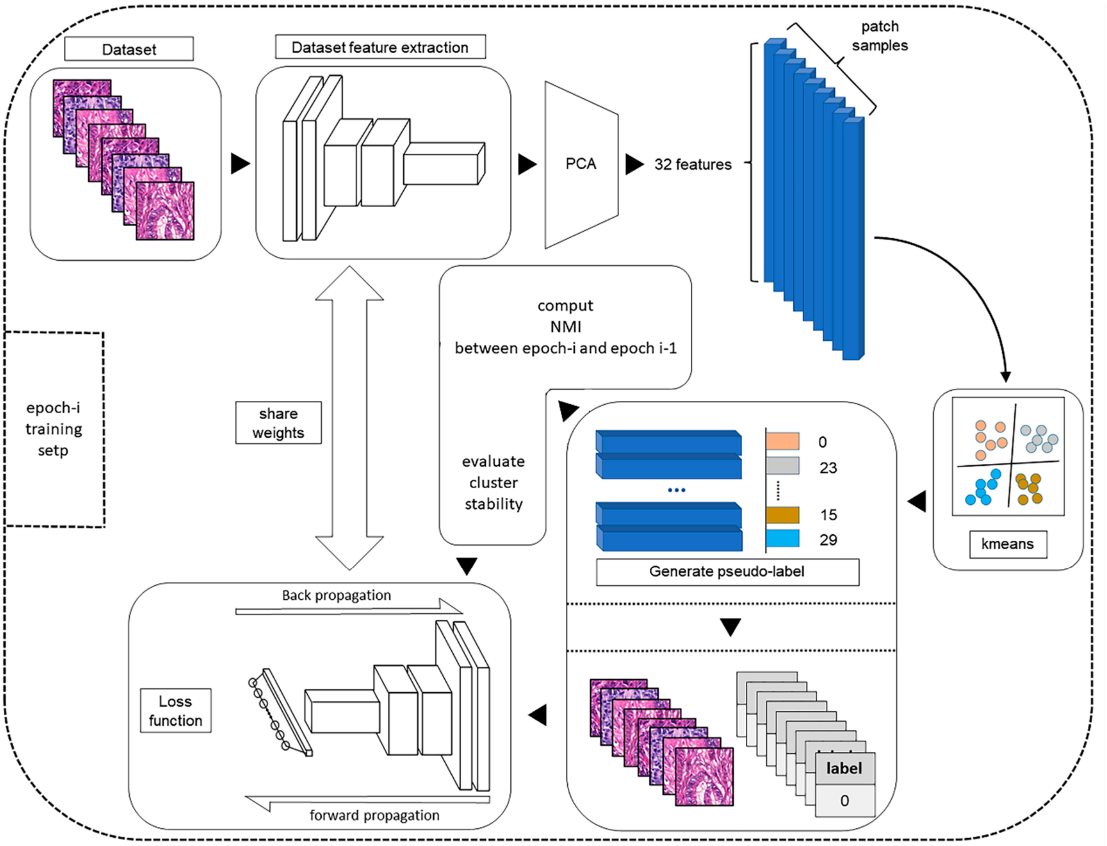

Figure S2. Visualization of the normalized mutual information (NMI) curves during the training process with different numbers of clusters from 5 to 50.

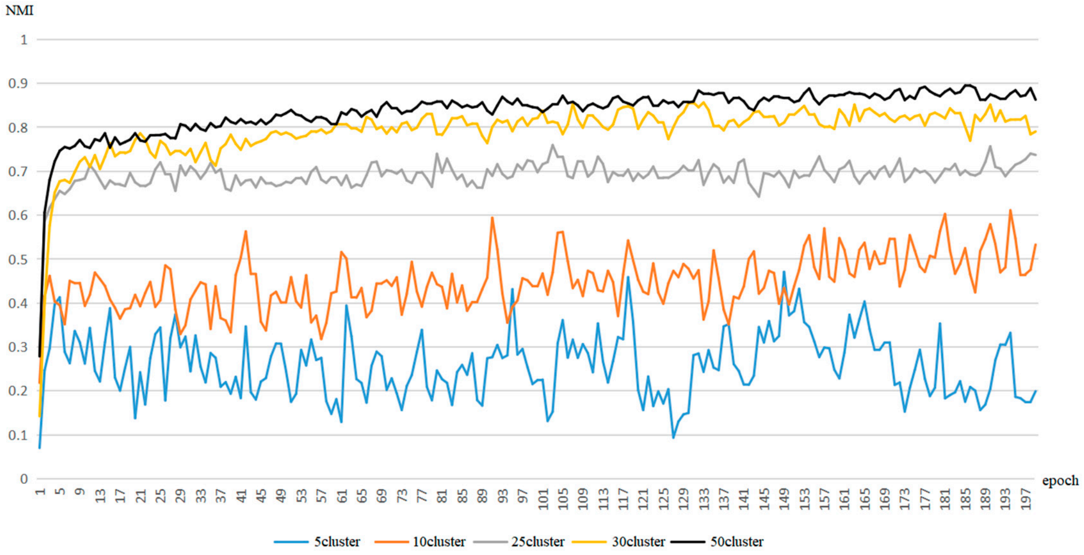

Figure S3. The normalized mutual information (NMI) curve during training. The maximum NMI was reached in epoch 132.

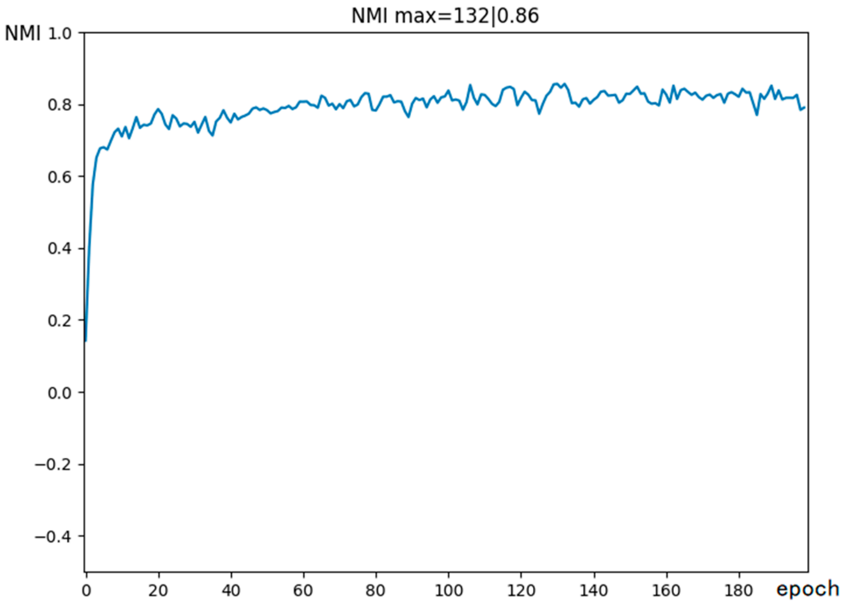

Figure S4. Schoenfeld residual test for the 3 significant clusters. The top is Schoenfeld residual test of Cluster13. The middle is Schoenfeld residual test of Cluster19. The bottom is Schoenfeld residual test of Cluster24. All of the P values for the three clusters in Schoenfeld residual test were less than 0.05, indicating that the clusters included in the Cox regression satisfy the proportional hazard assumption.

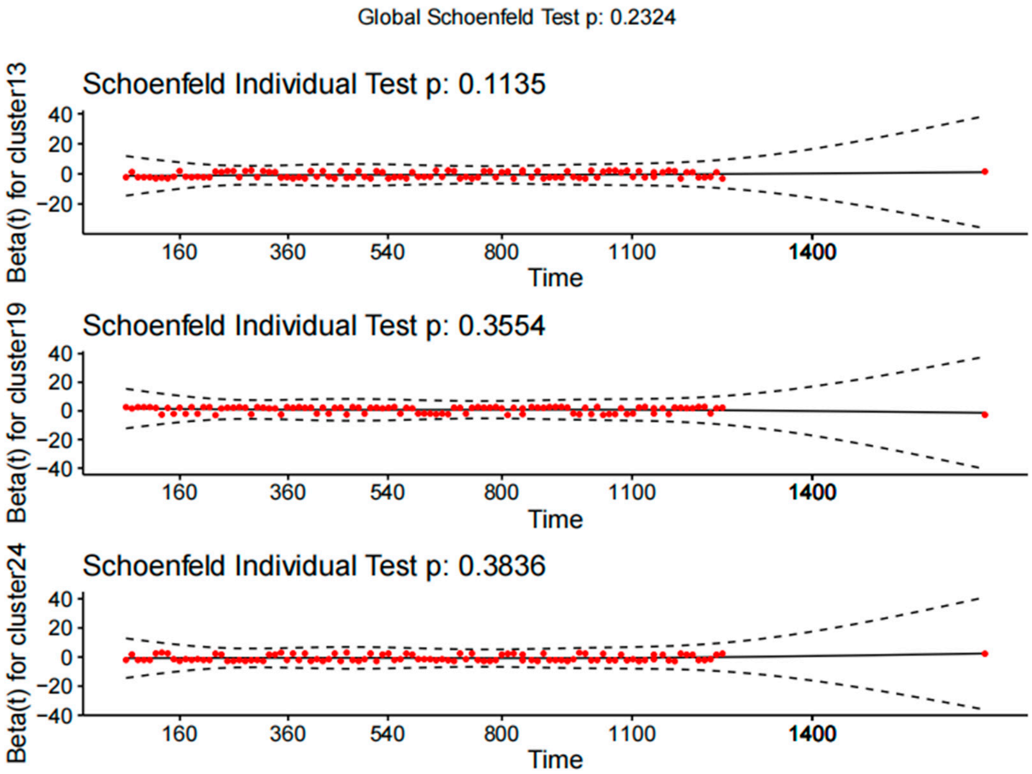

Supplement: Supplementary file 1 [file bioengineering-13-00334-s001.zip › Supplementary Figures.pdf]
